# Additional collection devices used in conjunction with the SurePath Liquid-Based Pap Test broom device do not enhance diagnostic utility

**DOI:** 10.1186/1472-6874-4-6

**Published:** 2004-09-13

**Authors:** Sarah J Day, Darla L O'Shaughnessy, Jason C O'Connor, Gregory G Freund

**Affiliations:** 1 Departments of Cytopathology, Carle Clinic Association, Urbana, IL, USA; 2 Obstetrics and Gynecology, Carle Clinic Association, Urbana, IL, USA

## Abstract

**Background:**

We have previously shown that use of an EC brush device in combination with the Rovers Cervex-Brush (SurePath broom) offered no significant improvement in EC recovery. Here we determine if use of additional collection devices enhance the diagnostic utility of the SurePath Pap for gynecologic cytology.

**Methods:**

After informed consent, 37 women ages 18–56 receiving their routine cervical examinations were randomized into four experimental groups. Each group was first sampled with the SurePath broom then immediately re-sampled with an additional collection device or devices. Group 1: Rover endocervix brush (n = 8). Group 2: Medscand CytoBrush Plus GT (n = 7). Group 3: Rover spatula + endocervix brush (n = 11). Group 4: Medscand spatula + CytoBrush Plus GT (n = 11).

**Results:**

Examination of SurePath broom-collected cytology yielded the following abnormal diagnoses: atypia (n = 2), LSIL (n = 5) and HSIL (n = 3). Comparison of these diagnoses to those obtained from paired samples using the additional collection devices showed that use of a second and or third device yielded no additional abnormal diagnoses. Importantly, use of additional devices did not improve upon the abnormal cell recovery of the SurePath broom and in 4/10 cases under-predicted or did not detect the SurePath broom-collected lesion as confirmed by cervical biopsy. Finally, in 36/37 cases, the SurePath broom successfully recovered ECs. Use of additional devices, in Group 3, augmented EC recovery to 37/37.

**Conclusions:**

Use of additional collection devices in conjunction with the SurePath broom did not enhance diagnostic utility of the SurePath Pap. A potential but not significant improvement in EC recovery might be seen with the use of three devices.

## Background

In gynecologic cytology, sampling of both the ecto and endocervix is critical to increasing Pap test sensitivity [1]. Controversy still exists, however, as to whether "all-in-one" broom-type devices appropriately sample the cervix. While agreement has been reached on the importance of sampling the transformation zone [2], concern as to how proximal the transformation zone is to the face of the cervix [3] has lead to lingering doubt over how effective current broom-type sampling devices are compared to a separate spatula and endocervical brush [4]. This debate has been re-energized by the generalized adoption of the liquid-based Pap test in the United States. Currently, two FDA approved liquid-based Pap tests are available, one manufactured by Cytyc (Boxborough, MA) and one manufactured by TriPath Care Technologies (Burlington, NC). Currently, the ThinPrep Pap Test (Cytyc) and the SurePath Pap (TriPath Care Technologies) offer two types of sampling devices a broom-type device or a spatula + cytobrush combination. It has been reported, that the use of the broom-type device for both the ThinPrep Pap Test and the SurePath Pap appears to under-sample the endocervix resulting in increased limited-bys due to lack of an EC component [4,5]. We have not observed this phenomenon and have previously shown that the SurePath Pap reduced by 33% the number of limited-by cases due to lack of an EC component when compared to the traditional Pap test when the SurePath Pap utilized the SurePath broom and the traditional Pap test utilized the spatula + EC brush combination [6]. In that failure to sample the endocervix can coincide with failure to sample the transformation zone we sought, in this study, to determine if additional sampling devices used in conjunction with the SurePath broom improved SurePath Pap EC recovery and/or increased SurePath Pap diagnostic effectiveness.

## Methods

### Cervical/endocervical sampling

After study design approval from the Carle Clinic Association Institutional Review Board and informed consent, 37 women ages 18–56 receiving their routine cervical examinations were sampled with the SurePath broom. This device, which is packaged with the SurePath Pap, is the Rovers Cervex-Brush (Rovers Medical Devices, Oss, The Netherlands). Its use followed the manufactures recommendations of 5 full clockwise rotations. The same patient was immediately re-sampled with either the Rovers endocervix brush, patient group 1; the Medscand Cytobrush Plus GT (Medscand Medical, Malmö, Sweden), patient group 2: the Rovers endocervix brush + Rovers Spatula, patient group 3; or the Medscand Cytobrush Plus GT + Medscand Pap Perfect Spatula, patient group 4. All devices had "pop-off" heads. The SurePath broom device was collected into CytoRich Preservative vials (TriPath Care Technologies, Burlington, NC) and processed routinely using the PrepStain Slide Processor (TriPath Care Technologies, Burlington, NC). In samples using multiple collection devices, all devices, including the SurePath broom, were placed in a single collection vial and processed as above.

### Slide diagnosis

The diagnostic terminology used was derived from the 1991 revision of the Bethesda System (TBS) [7-9]. The diagnostic categories available were: 1) no intraepithelial lesion (NIL), 2) inflammation/repair (BCC), 3) atypical squamous cells of uncertain significance (ASC-US), 4) low-grade squamous intraepithelial lesion (LSIL), 5) high-grade squamous intraepithelial lesion (HSIL) 6) squamous cancer, 7) atypical glandular cells of uncertain significance (AGUS) and 8) glandular cancer. ECs were considered present if they appeared as a group of 6 or more cells. Slides were reviewed following standard practice. Slide screening was performed blinded to the sample collection device type. Screening of alternate device collections from the same patient were screened by the same cytotechnologist. Screened slides were re-reviewed by a senior cytotechnologist and a pathologist for diagnoses other than NIL. Re-reviewers were blinded to device type and all slides from a particular patient were reviewed by the same senior cytotechnologist and pathologist. Quality control rescreen of slides did not result in revision of a diagnosis.

### Cytology/cervical biopsy comparison

All cases where the cytology diagnosis was ASC-US or more serious underwent colposcopic-guided cervical biopsy (cervical biopsy). Cases were excluded from analysis if the: 1) cytology and corresponding tissue diagnosis were separated in time by more than 6 months; 2) cytology and/or corresponding cervical biopsy were not performed and interpreted within the Carle Clinic Association/Hospital system. In cases where multiple cervical biopsies were performed, the cytology closest in temporal relationship to the cervical biopsy was correlated. No cervical biopsies met the exclusion criteria.

### Statistical analysis

All analyses were performed using SAS statistical software (Cary, NC). Data comparisons were made using the Student's paired t-test, the Sign Test and the Wilcoxon Signed Rank test for analysis of non-parametric data.

## Results

### Diagnostic utility of the SurePath broom with supplemental EC sampling

We have previously shown that use of the Surgipath C-E brush (Richmond, IL) in combination with the SurePath broom did not increase EC recovery in women undergoing a SurePath Pap [6]. This previous study, however, did not investigate whether use of an EC brush enhanced SurePath Pap diagnostic utility. Therefore, to determine if an EC brush aided recovery of cytologically abnormal cells, EC brushes from Rovers and Medscand were examined. Tables 1 and 2 show results of women who were first sampled with the SurePath broom then immediately re-sampled with either a Rovers (Table 1) or Medscand EC brush device (Table 2). In group 1 patients (Table 1), 1/8 had LSIL identified by cytology after use of the SurePath broom. In addition, EC cells were identified in all 8 cases. Immediate re-sampling of group 1 patients with the Rover brush did not result in increased abnormal diagnoses. In fact, 3/8 cases had less than 5000 squamous cells/slide and required a diagnosis of QNS. In these QNS cases, no EC cells were seen. Importantly, follow-up cervical biopsy confirmed the SurePath broom-identified LSIL. This case was associated with a Rovers brush QNS.

**Table 1:** Broom Followed By Rovers Endocervix Brush

**Broom Diagnosis**	**EC**	**Rovers Diagnosis**	**EC**	**Cervical Biopsy Diagnosis**
NIL	Yes	NIL	Yes	Ø
NIL	Yes	NIL	Yes	Ø
NIL	Yes	NIL	Yes	Ø
NIL	Yes	NIL	Yes	Ø
NIL	Yes	NIL	Yes	Ø
NIL	Yes	QNS	No	Ø
NIL	Yes	QNS	No	Ø
LSIL	Yes	QNS	No	LSIL

*EC, endocervical cells; **NIL**, negative for intraepithelial lesion; **QNS**, quantity not sufficient for diagnosis; **Ø**, no biopsy performed

P value for diagnosis: paired t test = 0.14, Sign Test = 0.25, Wilcoxon = 0.25

P value for presence of ECs: paired t test = 0.08, Sign Test = 0.25, Wilcoxon = 0.25

**Table 2:** Broom Followed By Medscand CytoBrush Plus GT

**Broom Diagnosis**	**EC**	**Medscand Diagnosis**	**EC**	**Biopsy Diagnosis**
NIL	Yes	NIL	Yes	Ø
NIL	Yes	NIL	Yes	Ø
NIL	Yes	NIL	Yes	Ø
NIL	Yes	NIL	No	Ø
ASC-US	Yes	ASC-US	Yes	NIL
HSIL	Yes	HSIL	Yes	HSIL
HSIL	Yes	HSIL	Yes	HSIL

*EC, endocervical cells; **NIL**, negative for intraepithelial lesion; **QNS**, quantity not sufficient for diagnosis; **Ø**, no biopsy performed

P value for diagnosis: paired t test = 1.0, Sign Test = 1.0, Wilcoxon = 1.0

P value for presence of ECs: paired t test = 0.36, Sign Test = 1.0, Wilcoxon = 1.0

When group 2 patients were examined by cytology after using the SurePath broom (Table 2), 1/7 patients had ASC-US and 2/7 had HSIL. As above, adequate EC cells were identified in all SurePath broom cases. Immediate re-sampling of group 2 patients with the Medscand brush did not provide additional diagnostic utility. Importantly, follow-up cervical biopsy confirmed the SurePath broom-identified HSILs. In one of these HSIL cases, the Medscand brush was associated with a QNS. Taken together these findings indicate that addition of a Rover endocervix brush or Medscand CytoBrush Plus GT to the SurePath broom does not improve SurePath Pap abnormal cell or EC recovery.

### Diagnostic utility of the SurePath broom with supplemental ectocervical and endocervical sampling

As shown above, use of an endocervical sampling device in addition to the SurePath broom did not enhance the usefulness of the SurePath Pap. To determine if a spatula plus an EC brush increased recovery of diagnostic cells over the SurePath broom, ectocervical spatulas from Rovers and Medscand were examined. Tables 3 and 4 show results of women who were first sampled with the SurePath broom then immediately re-sampled with either a Rovers spatula + endocervix brush (Table 3) or a Medscand PAP Perfect Spatula + CytoBrush Plus GT (Table 4). In group 3 patients (Table 3), 4/11 had abnormal cytology (1 AGUS, 3 LSIL) identified by use of the SurePath broom. EC cells were identified in 11/11 cases. Immediate re-sampling of group 3 patients using the Rovers devices resulted in 4 abnormal Pap diagnosis (2 ASC-US, 1 LSIL, 1 LSIL + AGUS) in the same four women. Complete diagnosis concordance, however, was seen in only one case. Follow-up cervical biopsy of these abnormals demonstrated 1 chronic cervicitis, 2 LSIL and 1 HSIL.

**Table 3:** Broom Followed By Rovers Endocervix Brush + Rovers Spatula

**Broom Diagnosis**	**EC**	**Rovers Diagnosis**	**EC**	**Cervical Biopsy Diagnosis**
NIL	Yes	NIL	Yes	Ø
NIL	Yes	NIL	Yes	Ø
NIL	Yes	NIL	Yes	Ø
NIL	Yes	NIL	Yes	Ø
NIL	Yes	NIL	Yes	Ø
NIL	Yes	NIL	Yes	Ø
NIL	Yes	NIL	Yes	Ø
AGUS	Yes	ASC-US	Yes	Chronic Cervicitis
LSIL	Yes	ASC-US	Yes	LSIL
LSIL	Yes	LSIL	Yes	LSIL
LSIL	Yes	LSIL, AGUS	Yes	HSIL

*EC, endocervical cells; **NIL**, negative for intraepithelial lesion; **Ø**, no biopsy performed

P value for diagnosis: paired t test = 0.34, Sign Test = 1.0, Wilcoxon = 1.0

P value for presence of ECs: paired t test = 1.0, Sign Test = 1.0, Wilcoxon = 1.0

**Table 4:** Broom Followed By Medscand CytoBrush Plus GT + Medscand Pap Perfect Spatula

**Broom Diagnosis**	**EC**	**Medscand Diagnosis**	**EC**	**Cervical Biopsy Diagnosis**
NIL	Yes	NIL	Yes	Ø
NIL	Yes	NIL	Yes	Ø
NIL	Yes	NIL	Yes	Ø
NIL	Yes	NIL	Yes	Ø
NIL	Yes	NIL	Yes	Ø
NIL	Yes	NIL	Yes	Ø
NIL	Yes	NIL	Yes	Ø
NIL	Yes	QNS	No	Ø
NIL	No	NIL	Yes	Ø
LSIL	Yes	ASC-US	Yes	LSIL
HSIL	Yes	ASC-US	Yes	HSIL

*EC, endocervical cells; **NIL**, negative for intraepithelial lesion; **QNS**, quantity not sufficient for diagnosis; **Ø**, no biopsy performed

P value for diagnosis: paired t test = 0.1, Sign Test = 0.25, Wilcoxon = 0.25

P value for presence of ECs: paired t test = 0.34, Sign Test = 1.0, Wilcoxon = 1.0

When group 4 patients were examined cytologically after sampling with the SurePath broom (Table 4), 1/11 patients had LSIL and 1/8 patients had HSIL. EC cells were identified in 10/11 patients. Immediate re-sampling of group 4 patients with the Medscand spatula + EC brush yielded 2 diagnoses of ASC-US and 1 QNS. EC cells were, again, identified in 10/11 patients, however, the patients lacking EC cells with the SurePath and Medscand devices were not concordant. Follow-up cervical biopsy confirmed the SurePath broom results as LSIL and HSIL. Taken together these findings indicate that addition of both a spatula plus a brush device does not alter SurePath Pap diagnoses but may enhance EC cell detection.

## Discussion

We have previously shown that the majority (88%) of SurePath Pap limited by diagnoses are due to lack of an EC component [6]. To overcome this problem, some clinicians have turned to using additional devices (usually an EC brush) in combination with the SurePath broom to attempt to increase the EC yield. The presumed rationale for use of an EC brush with the SurePath broom is that broom-type instruments do not reach into the cervical os as far as stand-alone brushes nor do they have bristles that are perpendicular to the handle. These concerns appear anecdotal but have concerned clinicians enough that TriPath Imaging sought and received recent approval for expanded labeling claims from the FDA to allow use of a spatula + brush combination with the SurePath Pap [10]. Our study was designed to test whether additional devices when used in combination with the SurePath broom enhanced recover of ECs or added diagnostic value to the SurePath Pap. The reason this study was undertaken was to demonstrate if the SurePath broom device was sufficient for obtaining an appropriate Pap sample. The strategy involved used a sequential testing method to show that added sampling of the cervix with spatula and/or brush devices did not recovery additional abnormal cells or additional ECs that altered the diagnostic results. These non-broom devices have different shapes and tinctoral qualities than the broom device, therefore, it is possible that they may sample portions of the cervix inaccessible to the broom, although no published evidence of such qualities exists. Importantly, this study was not designed to compare the SurePath broom to other devices used in the collection of SurePath Paps, instead, it was to designed to probe whether the SurePath broom alone was an appropriate device. This is important in light of the aforementioned SurePath Pap expanded labeling claim where clinicians might interpret such new FDA labeling as a repudiation of the broom device in favor of the spatula + brush combination. Currently, there is no available data detailing the results of this expanded labeling claim.

In this study, we found that 3% of women sampled with just the SurePath broom lacked EC cells. These findings were consistent with but better than our previous report based on 3,994 women in which we found that 6% of SurePath Paps were "satisfactory for evaluation but limited by no EC cells" [6]. Other studies using a broom-type device have reported a range of EC absence from as low as 4.38% to as high as 29.2% [4,11-16]. This considerable variability in EC recovery is not easily understood nor is it clear why in only one [11] of these seven other studies was the absence of ECs lower than our previously observed rate of 6%. The next lowest EC absence rate observed in these seven studies was 10.1% [13]. In our current investigation, the likely reason why our EC absence rate was lower than our previous findings [6] was the use of a single nurse practitioner to collect all samples. Important to the SurePath broom is the flat and rounded sides to each bristle. Counterclockwise rotation brings the rounded bristle edges in contact with the cervix instead of the flat side reducing device effectiveness. In addition, a single experienced collector is more likely to achieve a satisfactory Pap sample than multiple inexperienced Pap collectors [17]. Another important reason why our previous and current studies show a relative low EC absence rate is that these studies utilized liquid-based Pap preparation. Most previous studies focusing on broom-type devices have compared their effectiveness to other sampling devices using traditional preparation. As we have shown, the SurePath Pap reduces by 33% limited bys due to lack of ECs when compared to the traditional Pap [6].

Debate over cervical sampling devices often focuses on EC sampling. Unfortunately, few studies are available that report both the EC absence rate and the abnormal cell detection rate in studies where multiple devices are compared. The retrospective study by Boon et al [11] stands out in that it suggests that there is a correlation between lack of endocervical cell recover with the Rovers Cervex-Brush and reduced detection of CIN III. Most other studies have shown equivalence between spatula + EC brush and broom-type devices. In fact, Buntinx et al in a meta-analysis of 29 trials that included 85,000 patients concluded that there was no significant difference between spatula + cotton swab or EC brush, extended tip spatula or broom-type device in recovery of abnormal cells [18]. This analysis did underscore that use of just an EC brush, cotton swab or Ayre spatula alone is inappropriate. Interestingly, they also found that obtaining a second cervical sample immediately after the first, even with the same device, increased abnormal cell detection by nearly 33%. We, as Tables 1,2,3,4 demonstrate, did not see this benefit when using multiple devices. In the 37 patients we immediately re-sampled after use of the SurePath broom, no additional abnormal diagnoses were rendered nor was additional diagnostic material provided that clarified a SurePath broom collected indeterminate diagnosis.

As with any study, the strength of statistical analysis increases as the sample size is increased. However, even with small sample sizes, compelling results can be obtained if the statistical significance is large. Here the data was analyzed using three different nonparametric statistical tests (for non-continuous or non-numeric data) to ensure stringency. In addition, we chose to include the p-values for each of these statistical tests to show that multiple analyses yield the same result and that no single statistical test was chosen to favor a desired outcome. The hypothesis being tested, in this study, is that the use of additional collection devices in conjunction with the SurePath broom device does not enhance diagnostic utility. Normally, p-values <0.05 indicate that one should reject the hypothesis being tested and conclude enhanced utility. In this study, the large p-values generated from analysis of the data indicate a very high probability that the hypothesis be rejected and that no enhanced diagnostic utility is realized with the use of additional collection devices.

Since liquid-based Pap testing is relatively new, little work has been done to examine sampling device effectiveness utilizing this technology. Selvaggi et al compared the ThinPrep broom to the ThinPrep spatula + cytobrush and the ThinPrep broom +cytobrush [4]. These authors found that the EC component was missing in 24%, 10% and 13% of cases, respectively. However, no examination of diagnostic utility was included so it is not clear how these findings relate to device effectiveness in a liquid-based setting. In addition, their findings differed significantly from our previous examination of broom + brush combination using liquid-based preparation. When we examined 23 women for EC adequacy using both the SurePath broom and an EC brush, we found that the EC brush provided no additional benefit over the broom in the SurePath Pap [6]. Like the Selvaggi et al study we did not comment on diagnostic differences when a secondary device was added but unlike the Selvaggi et al study we found all broom-only samples to have EC cells present. Importantly, our current study is the first to examine diagnosis differences that may result from adding additional devices to a broom device in the liquid-based setting. Here we found that 10/37 (27%) of cases had abnormal cytology when the SurePath broom was used. Immediate re-sampling with a second or third device did not increase the number of abnormal cytologies found. In addition, cervical biopsy of all abnormal cytologies was performed and as Tables 1,2,3,4 show use of additional devices did not improve cytology/tissue correlation.

In conclusion, the SurePath broom appears to be a very effective cervix sampling device when coupled with the SurePath Pap. In 60 patients examined prospectively (37 in this study, 23 in our previous study [6]) only one patient (1.6%) failed to have EC cells recovered with the broom device alone. This is in contrast to the Selvaggi et al study that showed in 432 ThinPrep patients a 10% failure to detect ECs using two devices [4]. We must note, however, that the EC adequacy standard was different between their study and our studies because we defined EC presence as at least one group of 6 or more EC cells and they defined it as 10 or more EC and/or squamous metaplastic cells singly or in groups. Finally, our current work is the first to show in the liquid-based setting that the SurePath broom alone is as effective at identifying abnormal cells as the broom + additional devices.

## Conclusions

Use of additional collection devices in conjunction with the SurePath broom did not enhance diagnostic utility of the SurePath Pap. A potential but not significant improvement in EC recovery might be seen with the use of three devices.

## Competing interests

GGF has served as a speaker for TriPath Care Technologies.

## Abbreviations

Atypical squamous cells (ASC) of uncertain significance (ASC-US), endocervical cell (EC), Food and Drug Administration (FDA), high grade SIL (HSIL), low grade SIL (LSIL), Papanicolaou (Pap), quantity not sufficient for diagnosis (QNS), squamous intraepithelial lesion (SIL), SurePath Liquid-Based Pap Test (SurePath Pap), SurePath Liquid-Based Pap Test Broom Device (SurePath broom), The Bethesda System (TBS).

## Authors' contributions

SJD coordinated the study and analyzed the study data, DLO collected all samples, JCO performed the statistical analysis. GGF constructed the manuscript.

**Figure 1:**
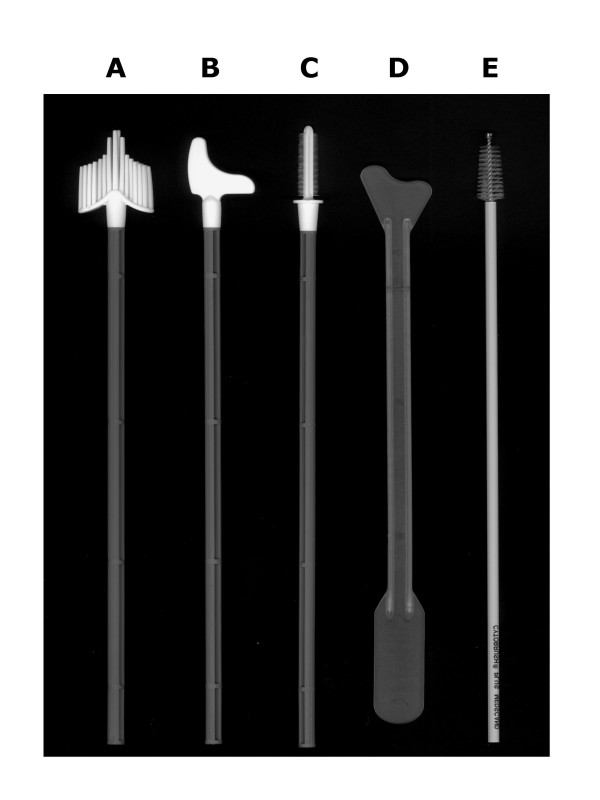
**Sampling Devices. ***A*, SurePath broom. *B*, Rover spatula. *C*, Rover endocervix brush. *D*, Medscand spatula. *E*, Medscand CytoBrush Plus GT.

## Pre-publication history

The pre-publication history for this paper can be accessed here:


